# Osteogenic Potential of Mouse Adipose-Derived Stem Cells Sorted for CD90 and CD105 In Vitro

**DOI:** 10.1155/2014/576358

**Published:** 2014-09-15

**Authors:** Maiko Yamamoto, Hidemi Nakata, Jia Hao, Joshua Chou, Shohei Kasugai, Shinji Kuroda

**Affiliations:** ^1^Oral Implantology and Regenerative Dental Medicine, Department of Oral Health Sciences, Graduate School of Medical and Dental Sciences, Tokyo Medical and Dental University, 1-5-45 Yushima, Bunkyo-ku, Tokyo 113-8510, Japan; ^2^Faculty of Science, University of Technology Sydney, P.O. Box 123, Broadway, Ultimo, Sydney, NSW 2007, Australia

## Abstract

Adipose tissue-derived stromal cells, termed ASCs, play an important role in regenerative applications. They resemble mesenchymal stem cells owing to their inexhaustibility, general differentiation potential, and plasticity and display a series of cell-specific and cluster-of-differentiation (CD) marker profiles similar to those of other somatic stem cells. Variations in phenotypes or differentiation are intimately associated with CD markers. The purpose of our study was to exhibit distinct populations of ASCs with differing characteristics for osteogenic differentiation. The primary cell batch of murine-derived ASCs was extracted from subcutaneous adipose tissue and the cells were sorted for the expression of the surface protein molecules CD90 and CD105 using flow cytometry. Each cell population sorted for CD90 and CD105 was analyzed for osteogenic potency after cell culture. The results suggested that ASCs exhibit distinct populations with differing characteristics for osteogenic differentiation: unsorted ASCs stimulated comparable mineralized nodule formation as bone marrow stromal cells (BMSCs) in osteogenic medium and viral transfection for BMP2 accelerated the formation of mineralized nodules in CD90 and/or CD105 positive ASCs with observation of decrease in CD105 expression after 14 days. Future studies assessing different immunophenotypes of ASCs should be undertaken to develop cell-based tissue engineering.

## 1. Introduction

Stem cells are characterized by their long-term self-renewal and potency across multiple lineages. Therefore, they have significant potential in tissue engineering and multidisciplinary regenerative medicine especially when they are assumed to be abundant and can be harvested via relatively invasive procedures such as adipose-derived stem cells extracted from the buccal fat pad compared with bone marrow stem cells. However, many of requirements such as establishment of ethic, safety and reproducibility still remain urgent before clinical use of stem cells, it is necessary, partially urgent, to pursue ideal stem cells so that they can then be differentiated along multiple cell lineage pathways in a regulated and reproducible manner, transplanted safely and effectively to an autologous or allogeneic host, and manufactured in accordance with current good manufacturing practice guidelines [1]. Recently, stem cell research has advanced rapidly with the application of human bone marrow and embryonic stem cells (ESCs) in regenerative medicine and repair although it is just at stages of basic research at this time. iPS cells can also be involved as another potential source for regenerative medicine. There is also a growing focus on the therapeutic use of adipose tissue-derived stromal cells, termed adipose-derived stem cells (ASCs), which play an important role in regenerative applications [2, 3].

Adipose tissue exists as both subcutaneous and visceral fat; subcutaneous fat is particularly easily aspirated and harvested during surgical operations. It contains several cell types including adipocytes, endothelial cells, fibroblasts, immune cells, vascular smooth muscle cells, and ASCs. ASCs are collected in the pelleted stromal vascular fraction (SVF) by centrifugation to separate them from the floating population of mature adipocytes. They resemble mesenchymal stem cells (MSCs) owing to their general differentiation potential and plasticity [4, 5] and display cell surface marker profiles similar to those of other somatic stem cells such as bone marrow-derived mesenchymal stem cells (BMSCs). ASCs express multiple stem cell-associated genes that are generally expressed by both BMSCs and ESCs. ASCs and BMSCs also both constitutively express genes that regulate angiogenesis, matrix remodeling, mitogenesis, and differentiation. Furthermore, both cell types express a series of cell-specific and cluster-of-differentiation (CD) markers [6–9].

Recent studies have revealed that multipotent ASCs, when cultured with the appropriate media supplements [1, 3], can be differentiated into the most mesenchymal cell types, including adipocytes [10–14], chondrocytes, osteoblasts [11–16], neuronal cells, myocytes, cardiomyocytes, hepatocytes, pancreatic cells, and endothelial cells although it remains unclear whether a single ASC could differentiate into all possible cell lineages. Even with these phenotypic differences, tissue repair was possible using freshly isolated SVF cells [17]. Variations in phenotypes or differentiation are intimately associated with cell surface markers such as the CD family. As such, identifying the ASC surface immunophenotype has allowed the stem cell population to be enriched and purified [18–20]. For example, the markers CD90 and CD105 can be used to identify the stem cells from total SVFs and adipose tissue likely to form osteoblastic or chondroblastic progenies [21]. CD105 has been identified as endoglin, an adhesion molecule that can distinguish adherent ASCs from hematopoietic lineages. However, only a small number of studies have examined the importance of CDs for stem cell growth and differentiation [3].

Many studies have demonstrated dramatic effects of BMPs on the osteoblastic differentiation of immature cells in vitro or bone healing in vivo in heterogeneous cell populations. Therefore, it was hypothesized that BMP2 as a substitute of the bone inducing BMP family members could be useful to assess or address osteogenic potential of CD-based, immunophenotypically sorted stem cells. In this study, murine-derived ASCs were sorted for the expression of the surface protein molecules CD90 and CD105 using flow cytometry. To our knowledge, this is the first study to examine the osteogenic potential of each sorted population with or without bone morphogenetic protein-2 (BMP2) adenoviral transfection. The aim of this study was to identify distinct populations of ASCs with differing characteristics for osteogenic differentiation.

## 2. Materials and Methods

The Institutional Animal Care and Use Committee of Tokyo Medical and Dental University approved the protocol design and procedures (approval number 0130286). Cell culture and molecular procedures were performed with the approval of and following the guidelines of Tokyo Medical and Dental University (approval number 2013-050A).

### 2.1. ASC Extraction

A total of 4.6 g of subcutaneous adipose tissue was extracted from the dorsal and ventral sides of 8-week-old female imprinting control region (ICR) mice, following pentobarbitone sodium euthanasia. It was then immediately digested into individual cells using 0.1% (w/v) collagenase (type I collagenase, Sigma-Aldrich, USA) in 30 mL phosphate-buffered saline (PBS, Wako Chemical, Japan) for 1.5 h. The cell suspension was filtered through a 70 *μ*m cell strainer (BD Biosciences, Japan) and then centrifuged at 1,000 ×g for 5 min to pellet the ASCs [4, 5, 22]. The cell pellet was resuspended in 50 mL Dulbecco's modified Eagle medium (DMEM, containing 4.5 g/L glucose and 0.584 g/L L-glutamine, Sigma-Aldrich) supplemented with 10% fetal bovine serum (FBS, Sigma-Aldrich) and 1% penicillin/streptomycin (Sigma-Aldrich) and plated on 10 cm culture dishes. Cells were incubated at 37°C in a humidified atmosphere consisting of 95% air and 5% CO_2_ until confluence was reached.

Mouse bone marrow cells were flushed out from femurs using a 27 G needle and pelleted quickly by centrifugation at 1,000 ×g for 5 min; they were then washed with PBS and centrifuged again. The harvested cells were cultured in 10 mL DMEM supplemented with 10% FBS and 1% penicillin/streptomycin on 10 cm culture dishes until they reached confluency. The media for both types of cells were refreshed every 3 days.

### 2.2. Flow Cytometry

The first passages of ASCs were trypsinized (1% trypsin-EDTA, Sigma-Aldrich) and then centrifuged at 1,000 ×g for 5 min in the presence of 1% FBS to quench the enzyme. The cell pellets were resuspended in 1% FBS in PBS, filtered through a 70 *μ*m cell strainer (BD Biosciences), and the cells were counted using a One Cell Counter (Wako Chemical). Twenty microliters of purified rat anti-mouse CD16/CD32 (Mouse BD Fc Block, BD Biosciences) was added to 2 × 10^7^ cells and the mixture was incubated at 4°C for 20 min. Cells were separated into 100 *μ*L fractions at a concentration of 1 × 10^7^ cells/mL and then incubated at 4°C with 0.5 *μ*g anti-CD90/Thy1 [FITC.MRC OX-7] (FITC) per 1,000,000 cells (ab226; Abcam, UK) and/or 10 *μ*L anti-CD105 antibody [MJ7/18] (phycoerythrin; ab93567, Abcam) per 1,000,000 cells for 40 min. Antibody conjugated cells were subsequently filtered again through a 35 *μ*m cell strainer (BD Biosciences). Cells were incubated with 5 *μ*L 7-AAD staining solution (BD Biosciences) per 10^6^ cells for 10 min and then sorted according to the expression of the cell surface markers CD90 and/or CD105 using fluorescence-activated cell sorting (FACS) (FACS Aria II; BD Biosciences, Japan). Cells were collected in DMEM supplemented with 1% penicillin/streptomycin and 10% FBS. ASCs were sorted into four groups: CD90(−)/CD105(−), CD90(+)/CD105(−), CD90(−)/CD105(+), and CD90(+)/CD105(+); unsorted ASCs were used as a control.

### 2.3. Microarrays

After flow cytometry using three 4.6 g-adipose tissue sets, total RNA was purified and collected from each of the four sorted cell populations, unsorted ASCs, and BMSCs using an RNeasy micro kit (Qiagen, USA). The RNA was then verified for quality and quantified using an Agilent 2100 Bioanalyzer and Agilent RNA 6000 Pico Kit (Agilent Technologies, Inc., USA). Two fragments associated with the 18S and 28S rRNAs were observed clearly, and the RNA integrity number of each sample was >8.0. The total RNA was then hybridized to an array containing 34,102 mRNAs and detection and quantification were then performed. The expression of marker genes associated with cell proliferation, adhesion, osteoblasts, osteoclasts, and Wnt signaling was then scanned, categorized, and represented in a heat map according to gene functions and/or signaling pathways (Cluster and Tree View; Stanford University, USA).

### 2.4. Preparation of an Adenovirus Encoding Human BMP2

**Figure 1:**
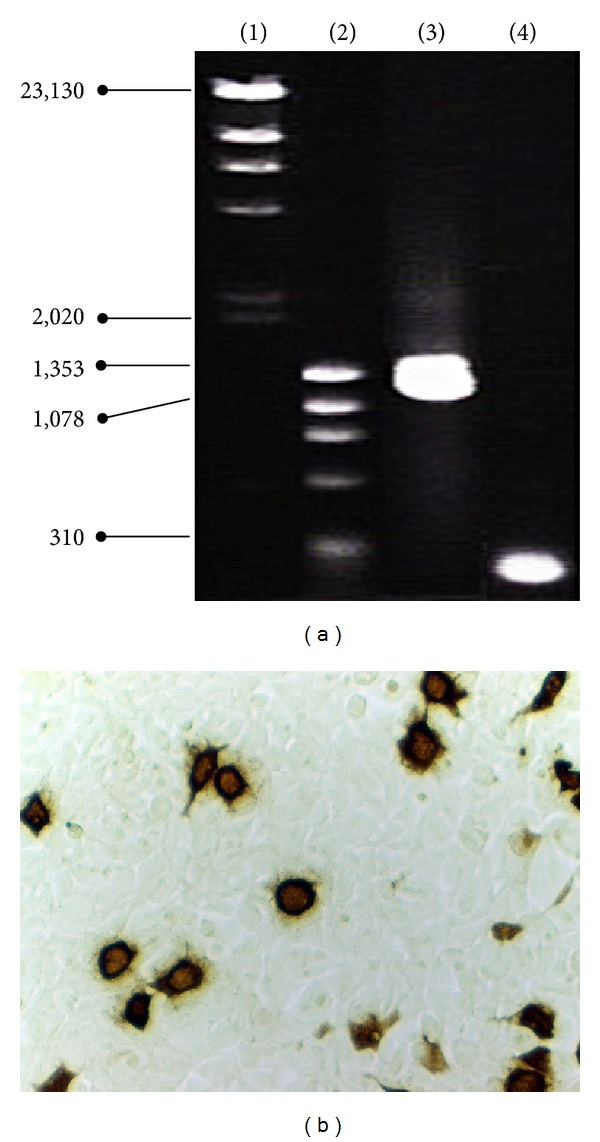
Definition of viral titers. (a) pAdenoX encoding human* bmp2* was identified using 2% agarose gel electrophoresis after PCR amplification with two primer sets. Lane 1: *λ*-*Hind*III digest; lane 2: φ X 174-*Hae*III digest; lane 3: full sequence human* bmp2* (1191 bp); lane 4: partial sequence human* bmp2* (225 bp). (b) HEK293 cells were infected with adenovirus encoding* bmp2*, which demonstrated a titer of 5.85 × 10^6^ ifu/mL.

The human BMP2 coding region (1,191 base pairs [bp]), which was cloned originally by Dr. Yutaka Maruoka [23], was ligated into the* Bam*HI restriction site of the pEGFP-N1 (Clontech Laboratories Inc., Palo Alto, CA, USA) vector; the vector had been amplified using an* E. coli* bacterial transformation system (TOP10F'; Invitrogen, Life Technologies, Japan). Clones were then purified and DNA was extracted using an EndoFree plasmid maxi kit (Qiagen). The full sequence of the* bmp2* gene was amplified using a primer set with 15 bp homology to each end of the multiple cloning site of the pAdenoX vector. The PCR product was then transferred into a pAdenoX vector using an infusion reaction (Adeno-X Adenoviral System 3, Clontech). The vector was amplified with Stellar competent cells and purified using an EndoFree plasmid maxi kit after PCR colony screening performed using the same primers (Figure 1(a)). Subsequently, the pAdenoX vector encoding human* bmp2* was linearized enzymatically with* Pac*I and transfected into HEK293 cells using chemical-based transfection (CalPhos Mammalian Transfection Kit; TaKaRa Bio, Japan). The cells then produced an adenovirus encoding human* bmp2*, termed AdenoX-bmp2, which caused cytopathic effects. The primary adenoviral stock was collected in PBS from three consecutive freeze-thaw cycles to prepare crude viral lysates. HEK293 cells were also retransfected with adenovirus by directly adding viral stock to the culture medium (2.5 × 10^5^ cells/500 *μ*L in each well, 1.9 cm^2^, of 24-well plate) to obtain higher viral titers (Adeno-X Rapid Titer Kit, TaKaRa Bio) (Figure 1(b)). After hexon major coat proteins were detected, the cells were fixed and incubated with hexon protein-specific antibodies. They were then incubated with HRP-conjugated antibodies and antigen-antibody complexes were visualized using 3,3′-diaminobenzidine substrate. Positive cells stained brown and could be easily counted under a 20× objective. The inclusion forming unit (ifu)/mL was calculated from the resulting mean number of positive cells/unit dilution. Different dilutions of adenovirus were obtained using the Adeno-X Rapid Titer method and used to infect HEK 293 cells. This resulted in a titer of 5.85 × 10^6^ ifu/mL. The secondary cytopathic adenoviral stocks were then stored at −20°C after the same freeze-thaw cycles.

### 2.5. Cell Culture

Cells sorted by FACS were seeded onto 24-well plates at an initial density of 4 × 10^4^ cells/well. They were then cultured for 14 days in normal DMEM supplemented with 10% FBS and 1% penicillin/streptomycin; in osteogenic medium supplemented with 10^−8 ^M dexamethasone, 10 mM *β*-glycerophosphate (G-9891, Sigma-Aldrich), and 50 ng/mL ascorbic acid (013-12061, Wako Chemical); or in BMP2 transfection medium, where the ASCs were transfected with AdenoX-bmp2 in osteogenic medium at a multiplicity of infection (MOI) of 5.85 × 10^6^ ifu/mL. Mock transfection was used as a control. ASCs from all groups were then cultured for up to 14 days. Unsorted ASCs and BMSCs were also treated as described. One set of cells was subjected to osteogenic analyses by measuring alkaline phosphatase (ALP) activity, assessing the protein expression of relevant genes, histological staining (ALP and mineralized-nodule staining), and reverse transcription polymerase chain reaction (RT-PCR). Another set was homogenized in TRIzol reagent (Invitrogen) to isolate total RNA for mRNA analyses and a third set was reanalyzed using FACS.

### 2.6. Quantifying Protein Concentrations and ALP Activity

Cells were cultured for 7 days, washed with PBS, scraped, lysed in 0.1% Triton X-100 (Sigma-Aldrich), and sonicated to disrupt the cell membranes. Samples were then centrifuged at 20,000 ×g for 10 min at 4°C and the supernatants were harvested to assess ALP activity and measure protein content. To determine protein concentrations, 100 *μ*L of prepared supernatant from each sample was mixed with 100 *μ*L of freshly prepared bicinchoninic acid (BCA) working reagent (QuantiPro BCA Protein Assay Kit, Sigma-Aldrich) and incubated at 37°C for 2 h. Samples were then quantified using a multilabel counter (MTP-650; Corona Electric, Japan) at a wavelength of 562 nm.

Quantitative and kinetic ALP activities were determined by assaying 50 *μ*L of sample supernatants using an ALP detection kit (Wako Chemical). Briefly, samples were incubated with* p*-nitrophenyl phosphate solution, which was prepared according to the manufacturer's instructions. The reaction was stopped by the addition of 50 *μ*L of NaOH stop solution, and ALP activity was measured using a multilabel counter (Wallac 1420 Arvo Sx, USA) at a wavelength of 405 nm. ALP activity was normalized to the total protein concentration in each sample. Data are expressed as means ± SD of three replicates.

### 2.7. ALP Staining

Naphthol AS-MX phosphatase (0.1 mg/mL) and Fast Blue BB Salt (0.6 mg/mL) were dissolved in Tris-HCl buffer (0.1 M, pH 8.8) containing 0.5% N,N-dimethylformamide and 2 mM MgCl_2_. The solution was then filtered and used as the ALP-positive cell staining solution (Sigma-Aldrich). Cells grown for 7 days were washed twice with PBS and fixed in 3.7% formalin for 10 min. Fixed cells were then rinsed with PBS twice and incubated in 1 mL staining solution at 37°C for 20 min to identify the blue ALP-positive cells. Cells were washed with PBS to stop the staining reaction. Digital images were captured using a microscope (Biozero BZ-8000; Keyence, USA) and the ALP-positive area was measured using Image J software (version 1.47, National Institutes of Health, USA) and calculated as the ratio of the positively stained area to the total area of the well.

### 2.8. Alizarin Red Staining

Mineralized nodules were stained after a 14-day culture in another set of wells. The staining solution was prepared by dissolving alizarin red S (1%) in 1 : 100 aluminum hydroxide in water, followed by filtration. Cells were washed twice with PBS and immersed in methanol for 10 min. After the cells were rinsed in water, they were incubated with 500 *μ*L of alizarin red S solution per well for 2 min until the mineralized nodules were stained red. The reactions were then terminated by washing with water to remove excessive staining precipitate and reagents. The images were captured by light microscopy (Biozero BZ-8000; Keyence).

To quantify the staining, cultures were destained using 10% cetylpyridinium chloride (CPC) in 10 mM sodium phosphate, pH 7.0, for 15 min at room temperature. Aliquots of extracts were diluted 10-fold in 10% CPC solution, and the concentration of alizarin red S was determined by measuring the absorbance at 562 nm on a multiplate reader (MTP-650, Corona Electric).

### 2.9. PCR for the Expression of Osteogenic Genes

The expression of type I collagen and osteocalcin, two genes related to osteogenesis, was analyzed by PCR using primer pairs designed using Primer 3 software as follows: mouse* collagen type I*—sense primer, 5′-CCCAGAGTGGAACAGCGATTAC-3′; antisense primer, 5′-TGTCTTGCCCCATTCATTTGTC-3′; mouse* osteocalcin*—sense primer, 5′–GCAATAAGGTAGTGAACAGACTCC-3′; antisense primer, 5′-GTTTGTAGGCGGTCTTCAAGC-3′; and mouse* GAPDH*—sense primer, 5′-CCACCCAGAAGACTGTGGAT-3′; antisense primer, 5′-CACATTGGGGGTAGGAACAC-3′. Cells were pooled and homogenized in TRIzol reagent at days 0 and 14 to extract total RNA, and cDNA was synthesized using the SuperScript First-Strand Synthesis System for RT-PCR (Invitrogen). The reverse-transcribed cDNAs were amplified with the appropriate primer sets under optimal cycling conditions: 35 cycles of 95°C for 60 s for denaturing, 60°C for 60 s for annealing, and 72°C for 60 s for extension. The identities of the PCR products were then confirmed using 2% agarose gel electrophoresis.

### 2.10. Statistical Analysis

Statistical analysis was performed using the statistic software package 14.0 SPSS for Windows (SPSS Inc. Chicago, IL). Data were analyzed using the nonparametric Tukey's HSD (honestly significant difference) test for group-by-group comparisons. Data were considered statistically significant when a *P* value was ≤ 0.05. Results are expressed as means ± standard deviations.

## 3. Results

### 3.1. Cell Expansion

**Figure 2:**
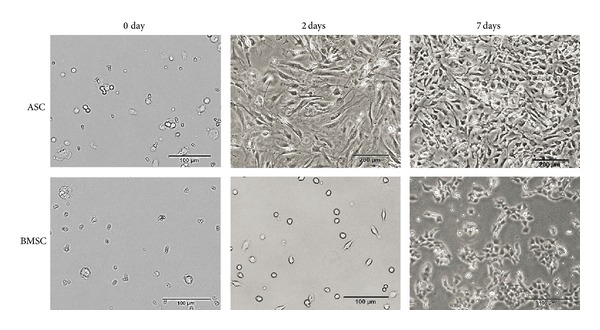
Expansion of ASCs and BMSCs. Cells from the second passage were cultured in 24-well plates at an initial cell number of 4 × 10^6^ cells/well for up to 10 days. The pictures show the discrepancy between the growing speed of ASCs and BMSCs. ASCs required 2 days to reach confluence, compared with 10 days for BMSCs.

Once they had been extracted from adipose tissue, it took 72 h for ASCs to reach confluence in 10 cm culture dishes; trypsin was then used to passage the cells. When cells reached confluence again, they were trypsinized and seeded (as the second passage) in three wells of a 24-well plate at a concentration of 4 × 10^4^ cells/well. These cells proliferated in DMEM and reached confluence by 48 h after seeding (Figure 2). The remaining trypsinized ASCs were used for cell sorting. BMSCs, which are heterogeneous population of fibroblasts, mesenchymal stem cells, hematopoietic cells, and many different progenitors, were cultured in growth medium to amplify adherent cells for 7 days. Medium with nonadherent cells was removed, and adherent cells were trypsinized and seeded into 10 cm culture dishes. The BMSCs grew slowly, taking 10 days to reach confluence. They were then seeded in three wells of a 24-well plate at an initial concentration of 4 × 10^4^ cells/well and allowed to proliferate. The BMSCs again grew slowly and reached confluence after 10 days of culture (Figure 2). The remaining trypsinized BMSCs were analyzed using flow cytometry.

### 3.2. FACS Analysis

**Figure 3:**
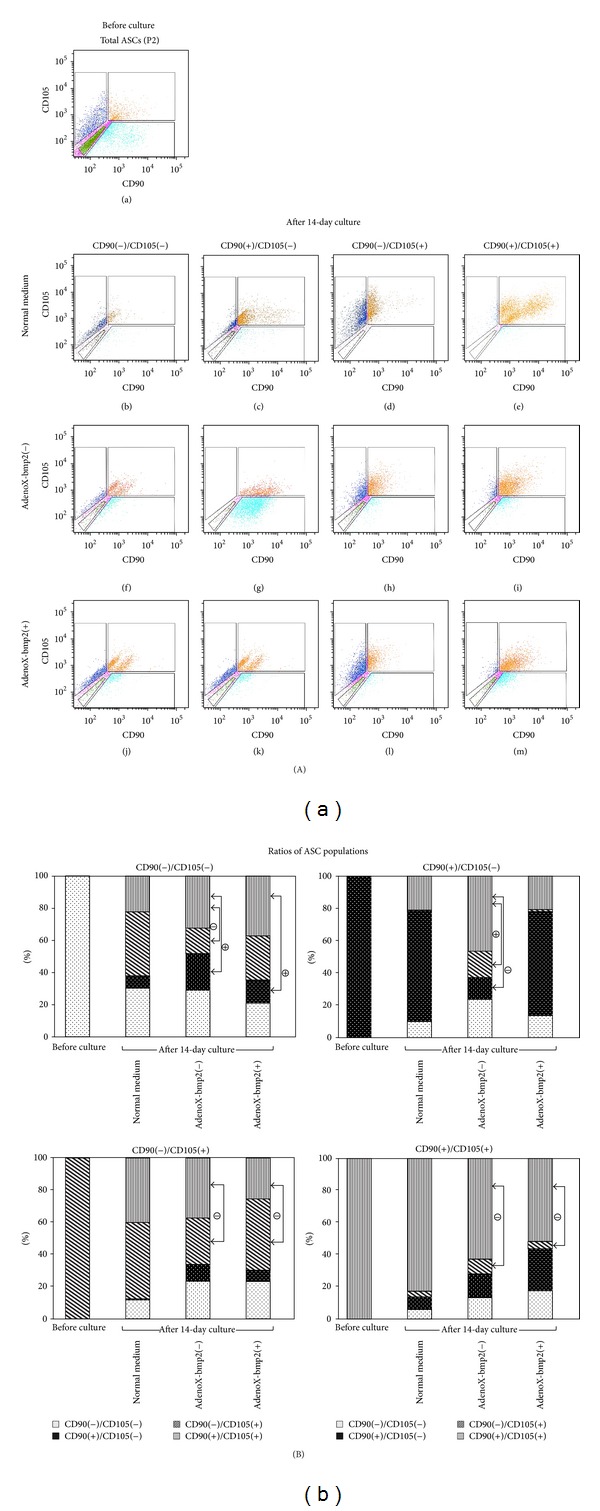
FACS analysis. (A) (a) ASCs at the second passage were separated into four populations: CD90(−)/CD105(−) (green), CD90(+)/CD105(−) (light blue), CD90(−)/CD105(+) (dark blue), and CD90(+)/CD105(+) (brown). ASCs pink in color were not categorized with any group. ((b)–(e)) ASCs were cultured in DMEM without osteogenic supplements (normal), ((f)–(i)) ASCs were cultured in osteogenic medium (AdenoX-bmp2(−)), whereas ((j)–(m)) ASCs were also transfected with AdenoX-bmp2 (AdenoX-bmp2(+)). (B) Ratios of the populations of the sorted ASCs altered in the three different culture media after 14-day culture, which resulted in changes of percentages of cells positive for CD90 or CD105: CD90(+)/CD105(−) plus CD90(+)/CD105(+) as CD90-positive, and CD90(−)/CD105(+) plus CD90(+)/CD105(+) as CD105-positive. The results are expressed as means of three replicates using three different batches of ASCs. Statistical significance was identified as follows: *⊖*, decreased compared to that in normal medium of 14-day culture; *⊕*, increased compared to that in normal medium of 14-day culture (*P* < 0.05).

Figure 3 shows the results of FACS analysis. FACS was used to separate the ASCs into four groups: CD90(+)/CD105(−), CD90(−)/CD105(+), CD90(+)/CD105(+), and CD90(−)/CD105(−). During this process, nonviable cells were eliminated using a cell viability assay (7-AAD cell viability assay kit; Life Technologies, USA). All the sorted cells survived in continuous culture for the experimental period of 14 days.

The expression patterns of membrane proteins were compared between groups at the end of the 14-day culture period (Figure 3). CD90(+)/CD105(−) cells expressed CD105 a little in DMEM without osteogenic supplements (normal medium); however, osteogenic medium elevated the number of CD105-positive cells, which was resumed by BMP2. In contrast, CD90(−)/CD105(+) cells began to express CD90 in the normal medium, whereas CD105 expression decreased in osteogenic medium and after subsequent BMP2 transfection. The CD90(+)/CD105(+) double-positive cell population expressed both markers more strongly during culture in the normal medium, whereas osteogenic medium and BMP2 transfection showed reduction of CD105 expression without changing CD90 levels. Finally, the CD90(−)/CD105(−) double-negative group altered its immunophenotype to CD90(+) and/or CD105(+) in all culture conditions. Interestingly, the initial ratio of the examined populations did not change statistically among all culture conditions in the unsorted ASCs (data not shown).

To summarize these results, the ASC markers CD90 and CD105 were induced during culture in DMEM without osteogenic supplements. In contrast, osteogenic medium and BMP2 transfection resulted in decrease of the expression of CD105. This suggests that CD105 might have some correlation to cell activities such as proliferation but not differentiation. However, the expression of CD90 and CD105 was observed in CD90(−)/CD105(−) and unsorted ASCs (data not shown) in all culture conditions, suggesting that the natural induction of CD90 and/or CD105 occurs during the culture of adipose-derived cells.

### 3.3. Genetic Clustering on a DNA Chip

**Figure 4:**
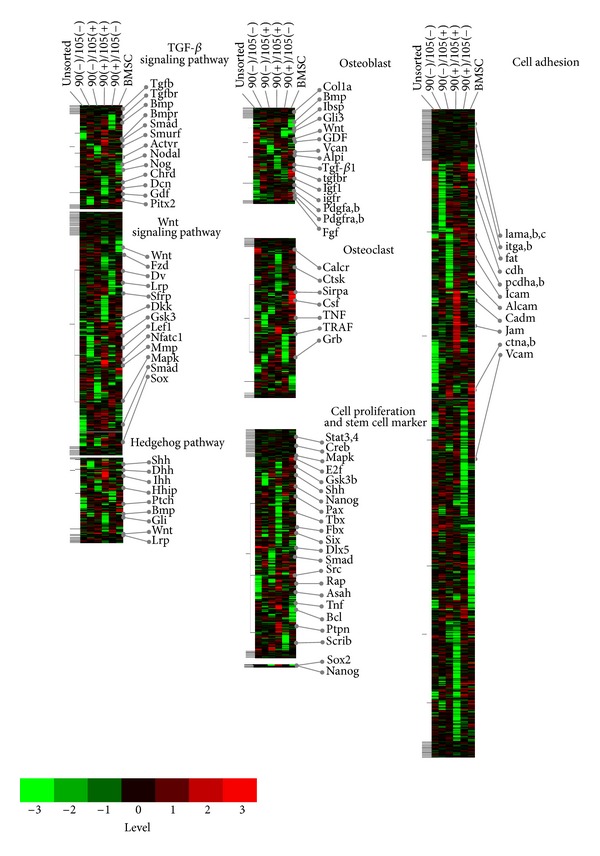
Microarray analysis. The expression levels of each gene were normalized among groups by the mean and then clustered into levels (−3 to +3) represented using colors as shown (Microarray Data Analysis Tool Ver. 3.2, Filgen, Japan).

The expression of 34,102 genes in each sorted population was profiled using DNA microarrays. Whole examined genes were categorized into groups based upon their biological roles and signaling pathways. Similarities in gene expression patterns were identified and are presented as a heat map (Figure 4, Table 2). Changes in the expression of bone-related genes were then analyzed in terms of proliferation, cell adhesion, osteoblasts, osteoclasts, and stemness. Growth factors were expressed slightly more in CD90(−)/CD105(+) cells, suggesting the acquisition of cell attachment due to CD105-stimulated cell proliferation. In contrast, many adhesion-related genes were expressed in CD90(+)/CD105(+) and BMSCs, whereas these genes were expressed only weakly in the CD90(−)/CD105(−) population. Osteoblastic genes were highly expressed in CD90(+)/CD105(−) and CD90(+)/CD105(+) cells, whereas BMSCs, which are representative of osteoblasts, expressed lower levels. Osteoclastic genes were expressed in BMSCs but to a much lesser extent than in all ASC groups. As shown in Figure 4, Table 2 robust expression of Wnt, which is associated with osteoblast differentiation, was detected in CD90(+)/CD105(+) cells and to a lesser extent in CD90(+)/CD105(−) and CD90(−)/CD105(+). In contrast, CD90(−)/CD105(−) and BMSCs did not express Wnt strongly.

### 3.4. The Numbers of Sorted Cells

**Figure 5:**
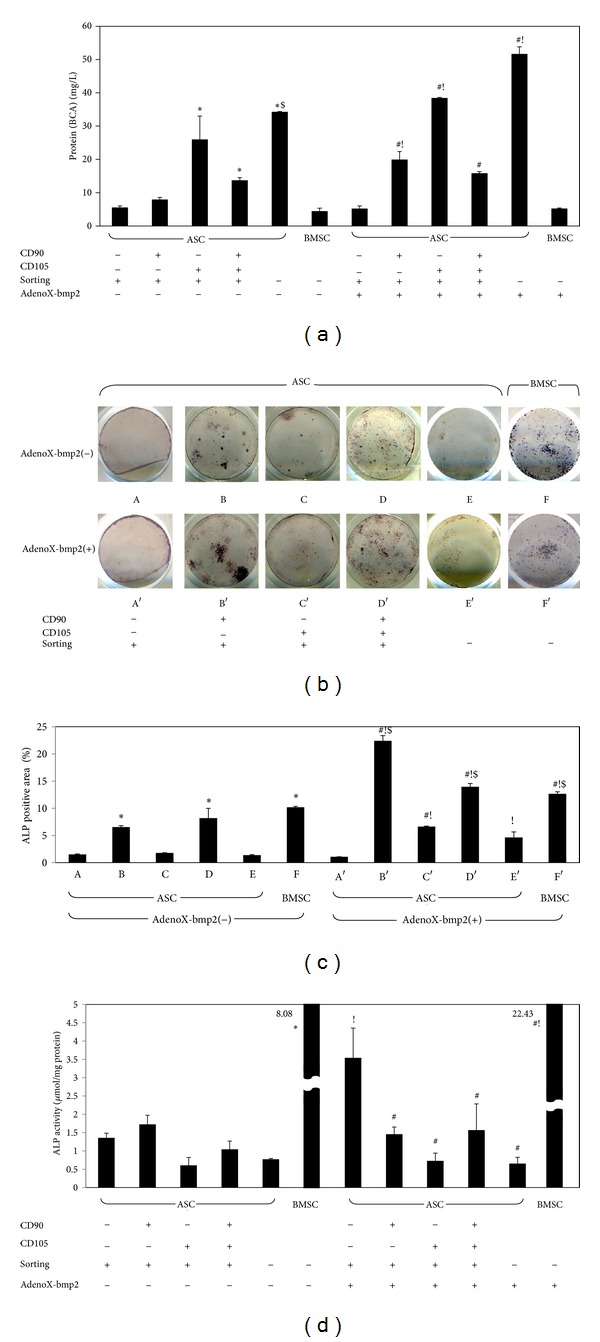
ALP activity. (a) CD90(−)/CD105(+) and unsorted ASCs contained higher protein concentrations. In contrast, CD90(−)/CD105(−) and BMSCs produced less protein in osteogenic conditions, even after BMP2 gene transfer. (b) ALP-positive staining (blue) appeared on day 7. CD90-positive ASCs and BMSCs (either with or without AdenoX-bmp2) showed an increased staining intensity. (c) The percentage of ALP positive area within the culture well was calculated. (d) Quantitative ALP activity was measured by normalizing total ALP amount to total protein. The results are expressed as means ± SD of three replicates. Statistical significance was identified as follows: ∗, versus A (CD90(−)/CD105(−), AdenoX-bmp2(−)); #, versus A′ (CD90(−)/CD105(−), AdenoX-bmp2(+)); !, within each population; and $, versus the others excluding populations B′, D′ and F′ (*P* < 0.05).

BCA protein assays revealed that CD105-positive cells, particularly CD90(−)/CD105(+), produced the highest concentration of protein after sorting, suggesting that the cell number of this population had increased to be the most. In contrast, the lowest protein concentration was detected in CD105-negative populations such as CD90(−)/CD105(−), suggesting the smallest increase in the population (Figure 5(a)). Protein concentration was statistically different between CD90(−)/CD105(−) and CD105-positive populations such as CD90(−)/CD105(+) and CD90(+)/CD105(+). BMP2 transfection increased protein concentration significantly, even in CD90(+)/CD105(−) cells, compared with CD90(−)/CD105(−) (Figure 5(a)). Furthermore, the number of BMSCs was fewer than all groups of sorted ASCs, with or without BMP2 transfection.

### 3.5. ALP-Positive Cells

Quantitative analysis of ALP staining in all groups was performed. The percentage of ALP positive area within the culture well was calculated. All ASCs, except for CD90(−)/CD105(−), and BMSCs showed a gradual and similar increase in the number of ALP-positive cells in culture over the 7-day experimental period (Figures 5(b) and 5(c)). When expression was compared between sorted and unsorted ASCs, ALP-positive cells were more prominent in CD90(+)/CD105(−), CD90(+)/CD105(+), and BMSCs with or without BMP2 transfection after 7 days of culture. Quantitative ALP activity was measured by normalizing total ALP amount to total protein. Strong ALP activity and concentrations were detected, even in CD90(−)/CD105(−) cells not transfected with BMP2 (Figure 5(d)). Although ALP activity was increased slightly by transfection with BMP2 in most groups, it was three-times higher only in CD90(−)/CD105(−) cells (Figure 5(d)), which might contribute to the very low protein concentration detected in these cells (Figure 5(a)).

### 3.6. Mineralized Nodules

**Figure 6:**
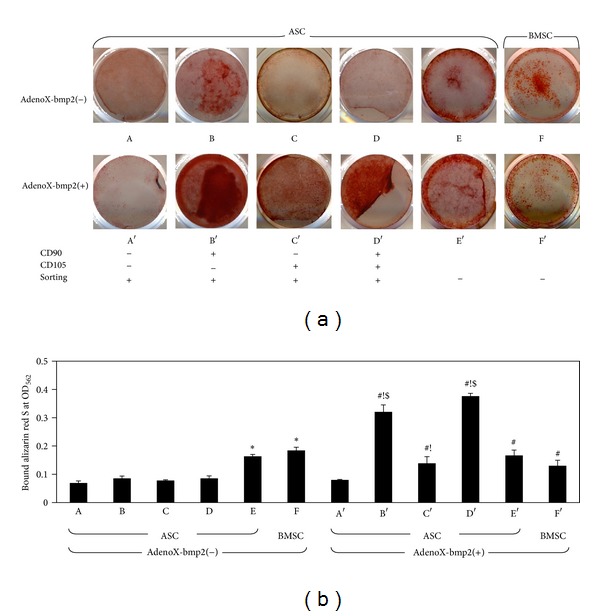
Mineralized nodule formation. (a) Calcium nodules (red) were observed using alizarin red S staining on day 14. (b) Quantitative analysis of alizarin red S staining was performed by measuring the absorbance of destained alizarin red S at 562 nm. The results are expressed as means ± SD of three replicates. Statistical significance was identified as follows: ∗, versus a (CD90(−)/CD105(−), AdenoX-bmp2(−)); #, versus A (CD90(−)/CD105(−), AdenoX-bmp2(+)); !, within each population; and $, versus the others excluding populations B and D (*P* < 0.05).

As shown in Figure 6, apparent mineralized nodules could be observed in CD90(+)/CD105(−) and unsorted ASCs without BMP2 as early as 14 days. However, BMP2 transfection increased mineralization in CD90(+)/CD105(−), slightly in CD90(−)/CD105(+), and particularly in CD90(+)/CD105(+). Interestingly, alizarin red S staining in unsorted ASCs, which deposited minerals at 14 days, was unchanged by BMP2 transfection, whereas CD90(−)/CD105(−) did not exhibit mineral adsorption, even after transfection. Nodule formation in BMSCs was reduced by BMP2, which might be explained by their reduced potential for propagation.

### 3.7. RT-PCR

**Figure 7:**
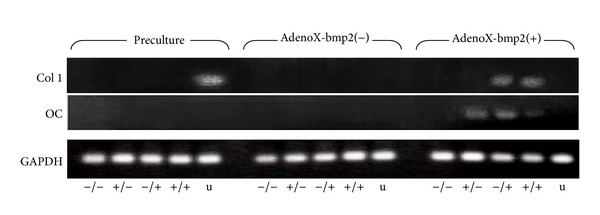
mRNA expression. PCR was performed immediately after FACS (preculture) and after 14 days of culture (osteogenic: AdenoX-bmp2(−), or BMP2 transfection: AdenoX-bmp2(+)). Collagen type I gene expression was stronger in CD90(−)/CD105(+) and CD90(+)/CD105(+) after AdenoX-bmp2 transfection. In contrast, a PCR product corresponding to* osteocalcin* was detected clearly in CD90(+)/CD105(−) and CD90(−)/CD105(+) after AdenoX-bmp2 transfection. The PCR cycling conditions were 35 cycles of 95°C for 60 s, 60°C for 60 s, and 72°C for 60 s. −/−, CD90(−)/CD105(−); +/−, CD90(+)/CD105(−); −/+, CD90(−)/CD105(+); +/+, CD90(+)/CD105(+); u, unsorted ASCs.

PCR products were separated and analyzed using 2% agarose gel electrophoresis (Figure 7). Osteocalcin, a noncollagenous protein found in bone and dentin, was used as a marker of calcium apposition. Therefore, we analyzed whether its expression was directly correlated with alizarin red S staining. The expression of collagen type 1 was stronger in CD90(−)/CD105(+) and CD90(+)/CD105(+) cells that had been transfected with BMP2. In contrast, a PCR product corresponding to osteocalcin was detected clearly in BMP2-transfected CD90(+)/CD105(−), CD90(−)/CD105(+), and CD90(+)/CD105(+) cell populations.

## 4. Discussion

To date, no single MSC-specific marker has been identified. Several minimum criteria have been proposed for human MSCs, such as the following: adherence to plastic; the expression of CD73, CD90, and CD105; the lack of CD31, CD34, and CD45 during culture; and the ability to differentiate into adipocytes, chondrocytes, and osteocytes [24]. Mouse MSCs are similar to their human counterparts, except that they can lack or be heterogeneous for both CD73 and CD90 [25, 26]. CD105/endoglin is a high affinity coreceptor for transforming growth factor (TGF)-*β*1 and TGF-*β*3 [27]. Although CD105 is generally considered to be an important MSC marker [24, 28], several reports have shown that its expression varies depending upon the source of MSCs (bone marrow, adipose tissue, umbilical cord blood, or placenta), the culture time in vitro, and differentiation status [29–32].

SVF from adipose tissue consists of mainly endothelial cells, hematopoietic cells, and ASCs. Most human ASCs are CD105(+), CD90(+), CD34(+), CD31(−), and DLK1(+) [21]. In contrast, endothelial cells are CD90(−), CD34(−), and CD31(+) [33], whereas the hematopoietic fraction is characterized as CD105(−) in murine cells [34]. In the present study, the isolation of ASCs negative for CD105 was the requirement for forming an osteogenic population. This was because previous studies revealed that CD105(−) ASCs exhibited enhanced adipogenic and osteogenic potential, probably due to reduced TGF-*β*/SMAD2 signaling [35, 36]. However, previous studies in humans demonstrated that CD105 was increased and highly expressed as early as the fifth passaging of ASCs [21, 37–39]. In the current study, most ASCs at the second passage were assumed to be CD105(+), CD90(+), CD34(+), and CD31(−). Therefore, we predicted that there was only a small population of CD105(−) ASCs in the SVF. However, CD90(−)/CD105(−) ASCs were the major population identified by FACS. A previous report suggested that there was a small but continuous reduction in CD105 protein expression during the expansion of murine ASCs when cultures became confluent, whereas CD105 mRNA levels increased or were downregulated during the passage of cells [40]. This supports our results obtained from PCR (data not shown) and FACS analyses describing changes in CD105 expression. Therefore, cell expansion during the passaging and cultivation of ASCs might interfere with the translation of CD105 in an autocrine or paracrine manner. Moreover, CD105 was predominantly localized in the transport compartments of ASCs, and only a small amount was found in the outer membrane. Although this might limit detection of the CD105 antigen, the present study clearly revealed a small population of CD105-positive cells using FACS analysis.

CD90 is an extracellular matrix molecule and a mesenchymal stem cell marker. Together with CD105, it plays a role in allowing cells to become immortal and multipotent [21]. It also plays a role in osteogenesis together with CD146, collagen types I and III, osteopontin, and osteonectin [1]. Therefore, CD90-positive ASCs have strong osteogenic capacity in vitro and in vivo [41]. In the current study, FACS analysis revealed that most ASCs expressed CD90 by the second passage. However, osteogenic staining revealed stronger staining in the CD90-positive groups compared with unsorted ASCs, suggesting the importance of this immunophenotype for the differentiation of stem cells into other cell types.

It was assumed that total protein amount tested in the study would be correlated to cell number since the subsets of CD105-positive populations producing increased protein examined here showed increase of summed cell cycle gene expressions demonstrated on the DNA chip (Figure 4, Table 2), which might imply cell proliferation; however, there still might be a leap in the logic and then total protein represented not so much cell proliferation activity as cell number and was just used for normalization of the osteogenic activities. CD90(+)/CD105(−) and CD90(+)/CD105(+) cells had strong osteogenic potential and exhibited mineralized nodules, whereas CD90(−)/CD105(+) encouraged total protein which might be indirectly associated with cell proliferation. This might reveal a potential role for CD90 and CD105 in stem cells [35, 36, 41]. Therefore, strong expression of CD105 might predict weak osteogenesis. However, stronger staining of ALP and mineralized nodules was observed in CD90(+)/CD105(+) than in CD90(−)/CD105(+) and CD90(−)/CD105(−) populations, suggesting proliferation due to the strong expression of CD105. This subsequently influenced CD90-stimulated differentiation over the experimental period of 14 days, during which CD105 attenuated. Interestingly, FACS analysis revealed that CD90 expression was unchanged over the study period. A previous study reported that rat-derived mesenchymal stem cells lose their ability for osteogenesis with increased passage [42]. This suggests that CD105 expression might have been maintained in this cell population, although this was not investigated.

Many reports have demonstrated dramatic effects of BMPs on osteogenesis, such as the osteoblastic differentiation of immature cells in vitro or bone healing in vivo in heterogeneous cell populations. However, only a small number of studies have assessed their role in multipotent CD-based, immunophenotypically sorted stem cells, particularly regarding osteoblastic differentiation [40]. Therefore, it remains unclear which types of cells are susceptible to BMP-induced differentiation. The present study was the first to infect CD-based immunophenotypically sorted stem cells with an adenovirus carrying BMP2. Generally, the osteogenic effects of media containing dexamethasone and BMPs on cultured cells have been assessed independently; therefore, their potential synergistic or reciprocal effects on osteogenesis have not yet been determined. In this study, we used osteogenic medium supplemented with dexamethasone to determine whether exogenous BMP2 accelerated osteogenesis in combination with osteogenic medium. Although no dramatic effects of BMP2 on ALP activity were seen after 7 days, a significant increase in the number of mineralized nodules was detected, except in CD90(−)/CD105(−) cells, after 14 days. This suggests that ALP activity had already been activated before 7 days and that BMP2 transfection increased the number of mineralized nodules overall without changing the relative number of mineralized nodules among groups. Interestingly, mineral deposition in unsorted ASCs was not affected by the viral transfection of BMP2. Taken together, these data suggest that nonstem-like cells, such as CD90(−)/CD105(−), might regulate stem cells in coculture. For example, AdenoX-bmp2 accelerated osteoblastic differentiation, particularly in CD90-positive ASCs. Therefore, BMP2 had a lesser effect on osteogenesis in CD90-negative populations such as CD90(−)/CD105(−).

Nevertheless, the appropriate dose of BMP2 for osteoblastic maturation or bone generation in vivo should be optimized. In the current study, a MOI of Adeno-bmp2 of 5.85 × 10^6^ ifu/mL was used for the viral transfection of ASCs in dexamethasone medium, which further increased the formation of mineralized nodules via the synergistic effects of osteogenic medium and BMP2. However, the positive or negative effects of AdenoX-bmp2 in various culture conditions must be assessed. In future studies, viral transfection should be performed in growth medium or examined together with the viral gene transfection of cytokines or growth factors to advance ASC-engineering biology.

In the present study, primary cells extracted from subcutaneous adipose tissue by centrifugation were termed ASCs, even though they contained many CD90(−) and CD105(−) cells that might not be identified as stem cells. Although methods for isolating ASCs have already been established [4, 5, 22], most cells in our pellets were CD90(−)/CD105(−). In addition, the expression patterns of this double-negative population, which exhibited slightly increased CD90 and CD105 expression after culture in normal medium, changed with significant enhancement in CD90 and reduction in CD105 both in osteogenic medium and in BMP2 transfection. Indeed, these nonstem cells did not form prominent mineralized nodules in either media. In addition, the mixed population of unsorted ASCs did not show as much mineralization as CD90-positive populations although significant to CD90(−)/CD105(−) cells; this was likely due to CD90(−)/CD105(−) cells interfering with the other cell populations in the unsorted ASCs.

The data reported in this study revealed distinct osteogenesis-related characteristics of immunophenotypically different ASCs. CD90, which is required for a population to be defined as stem cells, was necessary for osteoblastic differentiation, whereas CD105 and CD90 were important for both osteoblastic differentiation and expansion. Over the 14-day culture period, CD105 expression was reduced in the CD105-positive populations, such as CD90(−)/CD105(+) and CD90(+)/CD105(+), in osteogenic medium and after BMP2 transfection. Although CD90-positive and CD105-negative cells were considered to be ideal for osteogenesis during cell-based engineering [35, 36], our microarray analyses suggest that CD90(+)/CD105(+) ASCs might also be promising candidates. In addition, CD90(+)/CD105(−) and CD90(+)/CD105(+) populations prominently revealed the potential of BMP2 transfection to stimulate the formation of mineralized nodules. For example, BMP2 might allow expanding cells at an early passage or culture time to differentiate into osteoblasts, indirectly, with strong reduction of CD105 expression.

## 5. Conclusion

The osteogenic potency of ASCs differed among the populations (Table 1). In addition, stimulation of mineralized nodule formation by the unsorted ASCs was comparable to that by BMSCs in osteogenic medium, and BMP2 significantly increased the formation of mineralized nodules especially in CD90-positive populations, such as CD90(+)/CD105(−) and CD90(+)/CD105(+), with observation of decreased CD105 expression. Further studies assessing different immunophenotypes of ASCs should be performed to develop cell-based tissue engineering.

## Figures and Tables

**Table 1:** Summary of biological activities of the populations.

Population	CD90(−)/CD105(−)			CD90(+)/CD105(−)			CD90(−)/CD105(+)			CD90(+)/CD105(+)			Unsorted			BMSC		
Medium	Normal	Osteogenic		Normal	Osteogenic		Normal	Osteogenic		Normal	Osteogenic		Normal	Osteogenic		Normal	Osteogenic	
BMP2	−	−	+	−	−	+	−	−	+	−	−	+	−	−	+	−	−	+
ALP positive area		●	●		●●	●●●		●	●●		●●	●●●		●	●●	● (Data not shown)	●●	●●●
Mineralized nodule		●	●		●	●●●		●	●●		●	●●●		●●	●●		●●	●●
Col 1									●			●	● (Preculture)			N/A	N/A	N/A
OC						●			●			●				N/A	N/A	N/A

Each population of ASCs and BMSCs expressed different aspects in osteogenic characteristics after 14 days of culture. ●: detected; ●●: significantly increased to those in CD90(−)/CD105(−) cultured in the osteogenic medium without AdenoX-bmp2. ●●●: significantly increased to the others except ●●●. All cells cultured in DMEM without osteogenic supplements (normal) remained less osteogenic after the period.

**Table:** (a) Total expression levels of gene types related to cell adhesion

	Itga	Itgb	Cdh	Pcdh	Alcam/Vcam	Cadm	Lama,b

CD90(−)/CD105(−)	−16	−6	−26	−12	−3	1	−11
CD90(+)/CD105(−)	−1	3	−13	−6	−2	−8	7
CD90(−)/CD105(+)	−12	−5	−4	−6	−3	−2	−3
CD90(+)/CD105(+)	9	−4	12	7	−8	0	−12
Unsorted ASC	−13	−2	−30	−29	−5	−3	6
BMSC	7	1	6	−16	12	−2	−9

**Table:** (b) Total expression levels of gene types related to cell proliferation

	Cell cycle			Body patterning and development								Oncogene and tumor suppressor							Stem cell marker	
	stat	Creb	E2F	Shh	Pax	Hox	Fbx	Tbx	Dlx	Six	Fgf/Egf	Src	Rap	Asah	Bcl	Scrib	Ptpn	Tnf	Nanog	Sox2

CD90(−)/CD105(−)	−1	−3	−1	−3	2	−2	1	4	1	0	−3	0	0	−1	−2	0	−1	−3	−1	0
CD90(+)/CD105(−)	1	−1	−5	4	2	3	−5	−4	−2	−1	−1	−2	−1	1	1	−1	0	1	0	0
CD90(−)/CD105(+)	−3	−3	4	−2	2	3	−4	2	−1	−1	1	1	1	−1	−1	1	−1	−1	0	−3
CD90(+)/CD105(+)	−2	3	2	−2	−1	−2	−2	0	1	3	1	0	2	2	4	2	2	1	1	2
Unsorted ASC	1	−1	0	0	−2	0	−2	−2	−2	1	2	1	−1	0	−2	0	−1	−1	0	0
BMSC	0	−1	1	−3	1	0	1	−3	−5	−3	−3	−1	3	−3	−3	0	1	3	−1	−3

**Table:** (c) Total expression levels of gene types related to osteoblast signaling

	Col1a	Bmp	Tgf/Tgfr	Fgf/Fgfr	Igf/Igfr	Pdgf/Pdgfr	smad	Wnt	Dcn	Vcan

CD90(−)/CD105(−)	1	−2	−7	0	−5	2	0	−2	−2	−3
CD90(+)/CD105(−)	1	8	−9	−2	−4	−3	0	−3	1	1
CD90(−)/CD105(+)	−3	−16	−1	−5	−4	1	2	−2	−1	−1
CD90(+)/CD105(+)	3	−7	−5	3	2	0	−1	0	2	1
Unsorted ASC	−3	−4	2	−1	−5	−6	1	0	−4	−1
BMSC	0	3	−4	2	−3	−1	−1	−2	0	−4

**Table:** (d) Total expression levels of gene types related to osteoclast signaling

	Calcr	Ctsk	Sirpa	Csf	Tnf	Traf	Grb

CD90(−)/CD105(−)	−1	1	4	−2	−2	1	−1
CD90(+)/CD105(−)	−1	1	−4	0	1	−1	3
CD90(−)/CD105(+)	1	−1	0	1	−4	2	1
CD90(+)/CD105(+)	−2	−3	−2	−1	−1	1	−3
Unsorted ASC	1	−2	−5	0	1	−3	−2
BMSC	0	0	3	3	6	−1	2

**Table:** (f) Total expression levels of gene isoforms related to Wnt signaling

	Wnt	Fzd	Dv	Lrp	Sfrp	Dkk	Gsk3	Lef1	Nfat	Mapk	Smad	Sox

CD90(−)/CD105(−)	−14	−12	−2	3	−1	−2	0	0	−1	1	−6	2
CD90(+)/CD105(−)	−11	−4	−2	−2	0	3	2	0	−9	−4	1	0
CD90(−)/CD105(+)	−2	−2	−2	−1	−3	−6	2	3	10	−1	−3	−2
CD90(+)/CD105(+)	5	−2	−2	2	−1	5	−2	−2	−9	1	7	1
Unsorted ASC	−16	4	0	0	−1	−4	−3	−3	−2	1	−3	0
BMSC	−5	−3	2	−3	9	−6	0	−4	−4	−6	1	1

	Wnt families											

CD90(−)/CD105(−)	8b, 9b											
CD90(+)/CD105(−)	2b, 3, 4, 5b, 6											
CD90(−)/CD105(+)	2, 3a, 5, 5a, 9a, 9b											
CD90(+)/CD105(+)	3a, 5a, 5b, 6, 9b, 10a, 10b, 11											
Unsorted ASC	4											
BMSC	4, 6, 7											
